# A universal lesion detection method based on partially supervised learning

**DOI:** 10.3389/fphar.2023.1084155

**Published:** 2023-08-01

**Authors:** Xun Wang, Xin Shi, Xiangyu Meng, Zhiyuan Zhang, Chaogang Zhang

**Affiliations:** ^1^ Department of Computer Science and Technology, China University of Petroleum, Qingdao, Shandong, China; ^2^ High Performance Computer Research Center, University of Chinese Academy of Sciences, Beijing, China

**Keywords:** PSL, ULD, 3D model, medical image learning, neural network learning

## Abstract

Partially supervised learning (PSL) is urgently necessary to explore to construct an efficient universal lesion detection (ULD) segmentation model. An annotated dataset is crucial but hard to acquire because of too many Computed tomography (CT) images and the lack of professionals in computer-aided detection/diagnosis (CADe/CADx). To address this problem, we propose a novel loss function to reduce the proportion of negative anchors which is extremely likely to classify the lesion area (positive samples) as a negative bounding box, further leading to an unexpected performance. Before calculating loss, we generate a mask to intentionally choose fewer negative anchors which will backward wrongful loss to the network. During the process of loss calculation, we set a parameter to reduce the proportion of negative samples, and it significantly reduces the adverse effect of misclassification on the model. Our experiments are implemented in a 3D framework by feeding a partially annotated dataset named DeepLesion, a large-scale public dataset for universal lesion detection from CT. We implement a lot of experiments to choose the most suitable parameter, and the result shows that the proposed method has greatly improved the performance of a ULD detector. Our code can be obtained at https://github.com/PLuld0/PLuldl.

## 1 Introduction

Medical image learning (Zhou et al., 2021; Qiao et al., 2022) is developing rapidly based on the emergence of machine learning (Song et al., 2021a; Song et al., 2021b; Xie et al., 2021; Song et al., 2022a; Song et al., 2022b; Li et al., 2022; Wang et al., 2022) and neural network (Meng et al., 2021a; Meng et al., 2021b; Wang et al., 2021; Qiao et al., 2022), thereby dramatically assists radiologist alleviating workload during reading computed tomography (CT) (Meng et al., 2022) images in computer-aided detection/diagnosis (CADe/CADx) (Wang et al., 2022). Meanwhile, universal lesion detection (ULD) (Li et al., 2022) is an important topic to develop a universal or multicategory CADe/CADx 3D framework, which needs to feed an annotated dataset on computed tomography (CT) (Yan et al., 2019; Li et al., 2020; Li et al., 2021). However, an exactly annotated dataset is impossible to get because of expensive manual labeling costs with the increasing number of CT images as well as the long-tailed distribution of disease species. (Tang et al., 2019). Therefore, partially supervised learning (PSL) which allows unannotated areas to exist is urgently necessary to explore to construct an efficient segmentation model.

A range of ULD methods is proposed to address the challenging task. For example, Zhou et al. (2019) proposed a prior-aware neural network by introducing explicitly anatomical priors of abdominal organ sizes during the training process. Dong et al. (2022) classified datasets into the corresponding type in terms of organs. Further developed multi-head detector to solve the problem of a partial label, which need a complex procedure to verify CT images. Fang and Yan. (2020) designed a fresh new approach by integrating a pyramid structure to extract context information of features, as well as modifying the last layer of the network to have multiple branches to segment previous organs and then to detect lesion by every classified organ. Lyu et al. (2021) used an additional segmentation branch to find the suspicious lesion anchors thereby assisting the conventional detection branch to reduce the negative impacts.

At present, most of the relevant research on object detection work is based on completely annotated data, that is, fully supervised learning (FSL) (Wang et al., 2021). However, with the progress of medical technology and the development of science and technology, CT imaging is becoming getting higher resolution, and hundreds of CT scanning images are produced every time. Therefore, partial supervised learning (PSL) technology is urgently needed.

**FIGURE 1:**
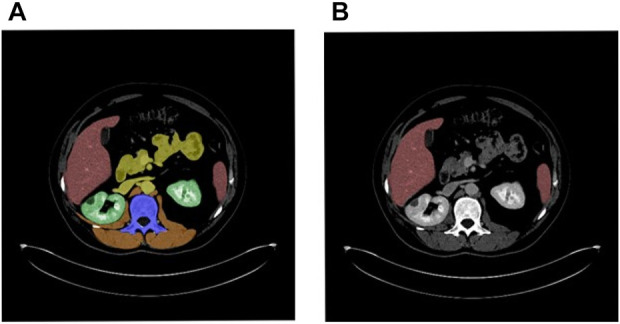
**(A)** FSL. **(B)** PSL. (Note that the picture is taken from Deeplesion. Masks are used only to illustrate concepts).

FSL (See Figure 1) which means all the samples are completely labeled, has developed rapidly thanks to the emergence of several well-known neural networks like RPN, Fast RCNN, Mask RCNN, and so on. Algorithms require feed to a fully labeled training dataset raised massively. Nair et al. (2020) developed a 3D lesion detection CNN to calculate lesion-wise uncertainties from voxel-wise uncertainties within detected lesions on the MS dataset. Cao et al. (2019) studied breast lesion diagnoses using a CNN network and evaluated their model in completely manually annotated images by experienced clinicians. Bria et al. (2020) proposed a two-stage deep learning framework to handle the extraordinary class imbalance that occurred during the training of small lesion datasets. The above studies are all conducted on specific diseases, such as gastric cancer, breast cancer, lymphatic cancer, etc. The types of diseases are fixed, and the organs with lesions are also certain.

**FIGURE 2:**
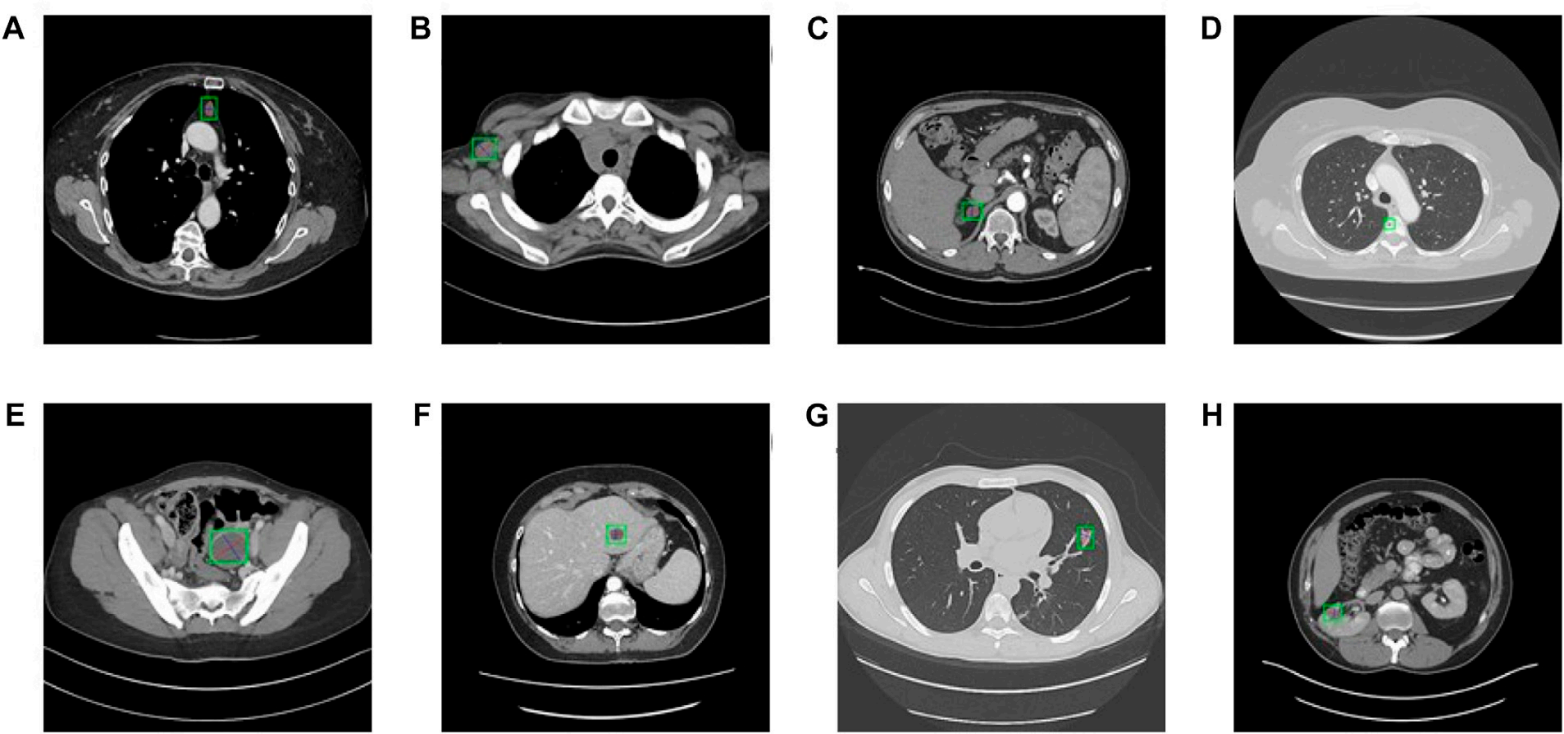
Eight types of lesions. **(A)** bone, **(B)** abdomen, **(C)** mediastinum, **(D)** liver, **(E)** lung, **(F)** kidney, **(G)** soft tissue, and **(H)** pelvis. Bookmarks whose size varies from 0.21 to 342.5 mm of various lesions are shown.

Concretely, the partial label issue is that only a part of the positive samples is labeled in the dataset of interest (see Figure 2). Currently, Lyu et al. (2020) proposed an identification algorithm to roughly minimize a risk estimator for the classification task, rather than lesion detection. Feng and Bo (2019) assist to maximize the semantic differences between two classes whose ground-truth are entirely different, further enlarging the difference of label confidences of two instances as well for a classification task. In conclusion, the current PSL algorithm mostly has been developed in the classification task. There are some detection and segmentation topics based on a small dataset or a specific lesion dataset. Therefore, the research of this paper is an innovative attempt in the ULD field.

All these methods proposed to deal with the partial label either process datasets classify according to the type of organs or lesions or develop a novel network that adds an extra branch to a conventional detection model. Compared to the above study, modifying the loss function is a simple and efficient method to reduce the influence of negative anchors. For instance, SUZUKI et al. (2022) proposed a loss function that uses a parameter to control the balance between two unlabeled positive samples and true negative samples. However, these methods are employed to models in a small dataset or a dataset that only contains one specific disease, which is unfriendly to those diseases that have few samples because rarely seen.

In this paper, we proposed a significant and simple method to modifying the loss function to solve the problem of misclassify unlabeled positive samples as negative samples, and our method is verified with a large dataset, DeepLesion, which concludes a large-scale public data for universal lesion detection from CT. Besides, our experiments are implemented in a 3D framework that is more powerful in capturing 3D context than 2D network although the latter benefit from large-scale a 2D pretraining. At present, the field of research using 2D convolution is very extensive. The algorithm proposed by Cao et al. (2023) improves a DNA storage encoding system with a graph convolutional network and self-attension. Before that, Cao et al. (2022) proposed a method to improve the DNA storage encoding system. Yin et al. (2021) also proposed the Marine Predator algorithm to solve the error rate in the process of DNA storage. Li et al. (2023) proposed to use 2D convolution learning information from Protein–protein interactions (PPIs). Sun et al. (2022) proposed a DNA triple design approach (TripDesign) based on interaction forces.

Our contribution to this work can be summarized as follows. Firstly, we generate a mask to intentionally choose fewer negative anchors which will backward wrongful loss to the network. Secondly, we set a parameter to reduce the proportion of negative samples, and it significantly reduces the adverse effect of misclassification on the model. Moreover, we implement a lot of experiments to choose the most suitable parameter, and the result shows that the proposed method has greatly improved the performance of a ULD detector.

## 2 Materials and methods

Partially supervised learning, or partial label, refers to one image containing one type of positive sample there may be other types of positive samples that are not labeled as positive. Nevertheless, these unlabeled positive samples still are treated as negative samples to feed to a conventional lesion detector. In our study, we trained the model in DeepLesion (Yan et al., 2018), which is a large-scale dataset of eight types of lesions (See Figure 2). Our method is employed to ignore the area where the unlabeled samples are located to decrease the effect of unlabeled samples. Our hypothesis is to magnify the ratio of positive samples and leave out the loss produced by unlabeled anchors, which will make further efforts to acquire more significant detection performance.

### 2.1 Negative anchor mask

In the process of generating ROI through the RPN network, the pixel-by-pixel mechanism is adopted to generate anchors on each pixel of the feature map according to the preset scale. Here, the scale size of anchors is usually set to (0.5, 1, 2). The generated anchors are expressed as (x, y, w, h), where x and y are the coordinates of the upper left corner of the anchors, w and h are the width and height of the anchors., IOU is compared with the ground truth according to the coordinates of the anchors and the size of the width and height. Referring to the threshold value, the upper limit of the threshold value in this study is 0.7, and the lower limit is 0.3. Therefore, anchors with IOU values higher than 0.7 are regarded as positive samples, and anchors with IOU values lower than 0.3 are negative samples. Thus, all anchors are gathered into an anchor vector, which is expressed as:

$$y={\left[0,0,0,1,1,1,0,1,\ldots ,0,1,1,0,1,0,0,0,0\right]}^{T}$$

where 1 represents positive samples, and 0 represents negative samples

In order to reduce the impact of mislabeled samples, we raise a mask produced based on normal distribution when backward loss of negative anchors during the region proposal process. Unlike models that have to classify datasets or design a complex network or execute models of different tasks in parallel mentioned in Section 2, our project only generates a mask that will neglect a partial negative bounding box so that enormously reduces the probability of misclassification. An outline of the proposed learning algorithm procedure refers to Algorithm 1. Specifically, the anchor vector executes a 1-label operation to obtain label_neg when calculating the negative samples, label_neg defined as:

$$y_{neg}={\left[1,1,1,0,0,0,1,0,\ldots ,1,0,0,1,0,1,1,1,1\right]}^{T}$$

After generating a mas, the proposed method successfully ignores some negative anchors and thus will greatly reduce the probability of transmission error loss of unlabeled lesions.

Algorithm 1 Generating Mask

**Input:**
Partially-labeled training dataset $D_{L}$ ;
Hyperparameters: λ ;
**Output:**
Detection model Θ

Input the dataset into the backbone network to extract features
Feed into feature pyramids structure to generate feature maps of different scales
Input to RPN network:
  Generate positive and negative anchors
  Compute mask
  Take λ and mask applied to negative anchors
  Generate region of interest
Train detection head
**Return** Θ

### 2.2 Negative proportion reduce factor

Furthermore, we separate the positive and negative anchors in the process of calculating the loss function of the RPN process, calculate their losses respectively, then pass them to the network. Our loss is modified according to the cross-entropy loss function, where the loss function of the positive samples is defined as:

$$L_{PS}=-\sum y_{i}∙\log p_{i}$$

where $y_{i}$ denotes the ground truth label of the anchor and $p_{i}$ is predicted label. There followed the loss of positive samples and we design a parameter to control the proportion of negative samples:

$$L_{NS}=-\lambda ∙\sum \left(1-y_{i}\right)∙\log\left(1-p_{i}\right)$$

There proposed method is essential due to the extreme imbalance of positive and negative samples that the number of positive labels in a ULD task is very little, even one CT image only contains one or two lesion ground-truth bounding boxes. Accordingly, the loss function added with the mask is defined as:

$$L_{M}=-\sum \left[y_{i}\log p_{i}+\lambda ∙M∙\left(1-y_{i}\right)\log\left(1-p_{i}\right)\right]$$

where *λ* denotes the added parameter, and M() is to manipulate the mask.

### 2.3 Fuse the proposed method into a 3D network

**FIGURE 3:**
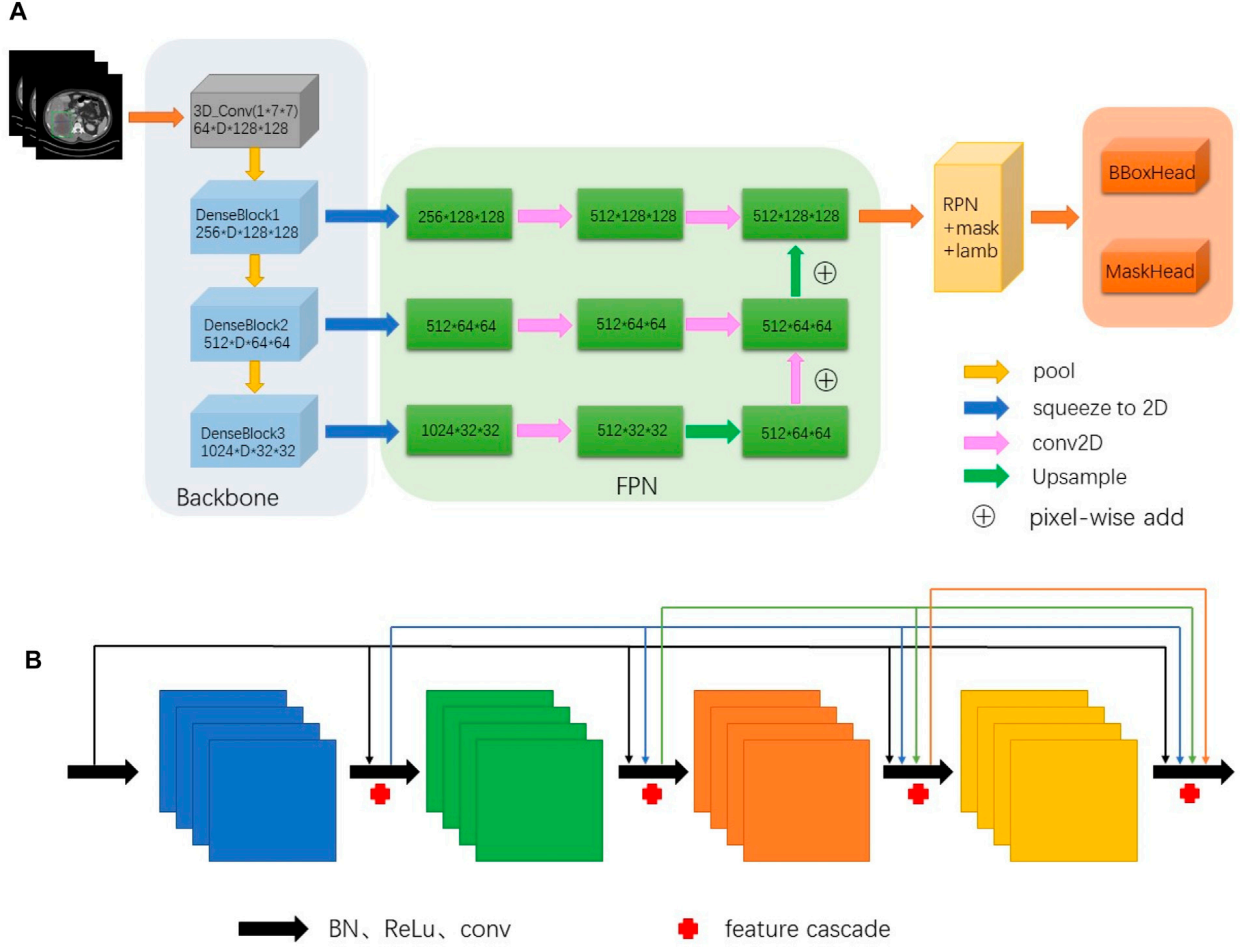
The structure of 3D model [above **(A)**] fused our method and backbone derived from DenseNet-121 [above **(B)**] takes a grey-scale 3D input of D × 512 × 512, where D is the number of slices (D = 7 in this study). Features from backbone are assembled and fused together in a feature pyramid network. Detection is based on segmentation framework using Mask R-CNN (He et al., 2017).

The proposed method can be introduced into any network of lesion-detection tasks. In our study, due to the dramatic performance of extracting context information from CT images, we decided to employ our method in a 3D extractor to detect lesions. For the structure of the 3D model refer see Figure 3. Detection is derived from instance segmentation framework Mask R-CNN, which backbone based on DenseNet-121 takes a grey-scale 3D input of D × 512 × 512, where D is the number of slices. Compared with 2D convolution, 3D convolution can effectively learn the spatiotemporal characteristics of continuous CT images. The filter of 2D convolution slides on the two dimensions of length and width, while the filter of 3D convolution needs to slide on the three dimensions of length, width and height. Therefore, when the filter slides across the entire 3D space, the output feature map is also 3D. In addition, 3D convolution is different from multi-channel convolution. 3D convolution preserves the spatial and temporal information of the input image, while 2D convolution can only output a feature map regardless of single-channel learning or multi-channel learning, thus losing the spatiotemporal information. 3D convolution is usually used in the research where the input data is video. In this study, because the training data image comes from continuous computer tomography, 3D convolution can preserve the spatiotemporal characteristics of the data and improve the network accuracy. In addition, fusing feature pyramids that feature from different scales are assembled during feature extraction. After input images pass through the feature pyramid, n feature maps of different scales are generated, and then they are fed to the RPN network to extract the region of interest. The feature enters the RPN network to generate a certain number of props (set to 2,000 in this experiment). Before that, the RPN network calculates the loss based on the anchor’s label and network prediction. Send the results back to the network and adjust the network parameters. The method proposed in this paper is integrated here. Finally, a 2D detection head is employed to detect lesions of key slices using the 2D feature map.

## 3 Results

### 3.1 Dataset and setting

The Deeplesion dataset includes 32,120 axial slices from 10,594 CT studies of 4,427 unique patients. There are one to three bookmarks in each slice, for a total of 32,735 bookmarks whose size varies from 0.21 to 342.5 mm of various lesions such as lung nodules, liver tumors, enlarged lymph nodes, and so on (See Figure 2). Most of the bookmarks that usually express critical lesion finds are measured in accordance with the response evaluation criteria in solid tumors (RECIST) handbooks. RECIST-diameter bookmarks indicate the concrete location and size of a lesion, is composed of two lines: one measuring the longest diameter of the lesion and another measuring its longest perpendicular diameter in the plane of measurement. Our study scaled the CT values from the intensity range (−1,024 to 3071HU) to the floating-point number in [0–255], which intensity covers the lungs, soft tissues, and bones. In addition, the size of each image slice is adjusted to 512 × 512. Because this research introduces a 3D network, we compose seven axial slices into a 7-channel image and feed it into the 3D network. The slice is a central slice containing bookmarks, and its adjacent slices are interpolated at 2 mm slice intervals. We only used horizontal flipping as an enhancement of training data and used random gradient descent (SGD) training for 200 epochs. The batchsize of the model is 2, and it takes about 7–8 h to train an epoch. The model converges at about 20 epoch, so the overall training time needs 4–5 days. The training equipment of the model is NVIDIA TITAN Xp 12 GB. The basic learning rate was set at 0.002, which dropped 10 times after the 12th and 14th epochs. The model using our method uses a lower positive anchor IOU threshold of 0.5, and other network settings are the same as the corresponding original model. We follow the official division, that is, 70% for training, 15% for verification, and 15% for testing. The number of false positives (FPPI) of each image was used as the evaluation index.

### 3.2 Experiments

**FIGURE 4:**
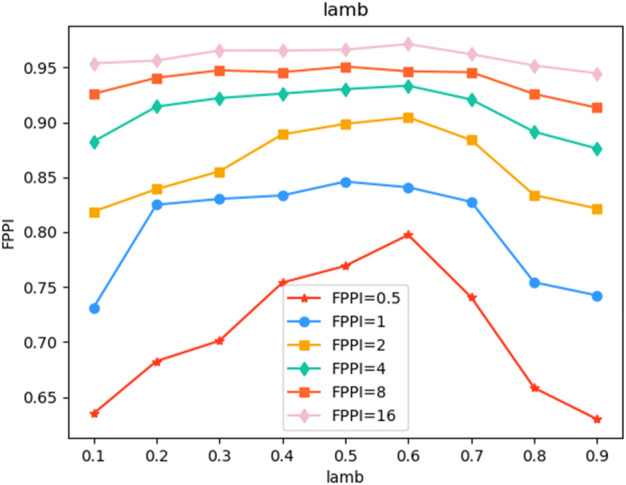
Trend of Model Performance with the change of parameter lamb.

Our experiment is mainly based on A3D. At the initial stage of the experiment, we set the learning rate to 0.02, and the problem of gradient explosion occurred in the network. Later, we conducted a lot of experiments on the value of the learning rate. When the learning rate was reduced to 1/10, that is, when the learning rate was 0.002, the model effect reached the optimum. When we were not sure of the above conclusions and lowered the learning rate to 1/10, the model convergence speed was very slow. Therefore, The learning rate set in the following experiments is 0.002. Figure 4 shows the influence of the negative sample proportion reduction factor *λ* When the accuracy of magic increases by 0.1 from 0 to 1, the model accuracy shows a trend of first increasing and then decreasing. When *λ* equals 0.1, the accuracy of the model is poor, which is caused by the extremely unbalanced number of positive and negative samples. When *λ* = 0.6, the performance of the model is optimal. After the mask is introduced, the model performance is shown in Table 1. We introduce the proposed method to the A3D model for performance comparison. As shown in the table, when slice = 7, that is, when the input image is 7D, the average FPPI value of this method is better than the A3D method of 0.82%. In addition, we also introduce the proposed method into the target detection algorithm Faster R-CNN and AlignShift, and the average performance is improved by 2.91% and 0.52% respectively, and 93.94% and 96.31% respectively in the best case. The model can achieve high accuracy because the proposed method can effectively improve the problem of partial labeling, and the proposed loss function can also improve the phenomenon of uneven distribution of positive and negative samples. In addition, in the experimental results, when FPPI = 0.5, the experimental results are most meaningful, and when FPPI = 16, the reference value of the results is not very significant, but the improvement of the model performance is beyond doubt.

**TABLE 1:** Sensitivity (%) at various FPPI on the testing dataset of Deeplesion.

Methods	Sclices	0.5	1	2	4	8	16	Avg. [0.5,1,2,4]
Faster R-CNN	3	56.83	67.66	73.92	82.15	90.23	92.26	70.14
Faster R-CNN (with ours)	3	60.44	70.60	75.83	85.32	91.55	93.94	73.05
AlignShift	7	76.88	83.24	89.15	92.74	93.17	95.68	85.50
AlignShift (with ours)	7	77.18	83.98	89.69	93.21	93.33	96.31	86.02
A3D	7	78.52	83.43	89.39	92.96	94.06	96.72	86.08
A3D (with ours)	7	79.74	84.08	90.46	93.34	94.65	97.13	86.90

### 3.3 Ablation study

We conducted ablation experiments on two key components of the proposed method. For example, in the first stage, we only introduced a negative sample proportion reduction factor without using the mask, and in the second stage, we used the mask without using a negative sample proportion reduction factor. The two experimental settings were compared. As shown in Table 2, when FPPI = 0.5 and 1, we achieved 0.41% and 0.33% improvement over the A3D baseline for the network that only introduced a negative sample proportion reduction factor. After only adding the mask, the performance is improved by 1.54% and 0.50% respectively. The best performance can be achieved by using the negative sample proportion reduction factor and mask at the same time. In addition, the convergence time of the model is greatly shortened.

**TABLE 2:** Ablation study of our method at various FPPI.

Mask	Factor	FPPI = 0.5	FPPI = 1
		78.52	83.43
**√**		80.06	83.93
	**√**	78.73	83.76
**√**	**√**	79.74	84.08

## 4 Discussion

In this paper, we proposed a novel loss function that introduces a negative sample proportion reduction factor and mask strategy exerted to improve the imbalance issue of anchors. The test dataset officially divided by Deeplesion is fed into the network for testing, and the results show that the proposed method can improve detection performance more strikingly than the existing partially supervised learning methods in the case of incomplete labeling. Compared with previous methods, this method effectively reduces the probability of backward error loss of positive samples. The method mentioned in this study can be applied to all those situations in that samples are classified by one hot coding that appears as a class imbalance. From this perspective, our research provides a new direction for the research of partially supervised learning.

However, the proposed method still exists some problems affecting detection performance. On the one hand, the same part has different labels. For example, one tissue is marked as soft tissue in one case but is marked as an abdomen in another case Our task does not require refining the types of lesions, but only finding the location of lesions. Therefore, the dataset may need to be further improved if a classification task of lesions is required in the future. On the other hand, the performance improvement of the model is limited because of the annotation of datasets, some image annotations are not pathological regions but only frame a certain organ or tissue. In this case, the model will backward the wrong loss, thus confusing the network in the training process. From this perspective, in future research, we will focus on autonomous power to identify whether it is a lesion of the network. Some incorrect samples are automatically discarded in the process of model training. In addition, this method is based on the cross-entropy loss function. In future research, we will expand the proposed method to other loss functions and hope to be applied to other networks to further improve detection performance.

## Data Availability

Publicly available datasets were analyzed in this study. This data can be found here: https://nihcc.box.com/v/DeepLesion.
